# Influences of persistent overweight on perceptual-motor proficiency of primary school children: the North-West CHILD longitudinal study: Persistent overweight and perceptual-motor proficiency in children

**DOI:** 10.1186/s12887-021-02708-x

**Published:** 2021-05-20

**Authors:** Elna de Waal, Anita Elizabeth Pienaar

**Affiliations:** 1 Physical Activity, Sport and Recreation (PhASRec), Focus area, Faculty Health Sciences, Potchefstroom Campus, North-West University, Private bag X 6001, Potchefstroom, 2523 Republic of South Africa; 2 Department of Exercise and Sport Sciences, School of Health and Rehabilitation Sciences, Faculty of Health Sciences, University of the Free State, Bloemfontein, 9301 Republic of South Africa

**Keywords:** Obesity, Overweight, Perceptual-motor proficiency, Motor skills, Early childhood

## Abstract

**Background:**

Overweight can be a precursor of poor motor execution, negatively impacting the overall development of school-aged children on various levels. This study determined the long-term influences of overweight on perceptual-motor proficiency (PMP) of primary school children in the North-West Province of South Africa.

**Methods:**

The study, which included 381 participants, formed part of the NW-CHILD longitudinal study from Grade 1 (6.86 years, ± 0.39) in 2010 to Grade 7 (12.9 years, ± 0.38) in 2016. Socioeconomic categories, called quintiles, were used to compare high and low socioeconomic status groups. Overweight was identified when BMI values fell above the 85th percentile, using age- and gender-specific cut-off points. The group was categorised into 4 BMI groups (never overweight, persistent overweight, overweight-to-normal and normal-to-overweight). The *Bruininks Oseretsky Test of Motor Proficiency-2 (BOT-2) Short Form* and selected composites measuring strength, balance, and running speed and agility were used to assess PMP.

**Results:**

A repeated measures ANOVA analysis indicated specific PMP differences between different BMI groups, but not total motor proficiency (*BOT-2 Short Form* total) differences. Practical significant group differences (Cohen’s d-values) were found in running speed and agility, strength, balance and the *BOT-2* total, over seven primary school years. The persistent overweight group showed the poorest performance over time, but also showed deteriorating skills and a gradual widening in perceptual-motor skills performance, compared to the other groups. BMI-related differences in perceptual-motor coordination were also more pronounced in older age groups.

**Conclusions:**

Persistent overweight negatively impacts specific motor-proficiency components, while improving weight status can counter these effects, which highlights the importance of timeous intervention to combat obesity at a young age.

## Background

Worldwide, alarming percentages of children (approximately 40 million under the age of 5 years) are overweight or obese with an increasing prevalence reported in most countries [1]. Since the year 2000, a 24% increase in the number of overweight children in Africa under 5 years has been reported [1]. Although some children outgrow obesity, a larger number display persistent overweight with lifelong developmental and health consequences [2–6]. Serious health risk factors are associated with obesity [2, 4], which necessitate finding solutions to combat overweight and obesity in children. Proficiency in motor skills is considered to be key in the development of young children as it shows strong influences on various developmental aspects, including healthy behaviour such as improved physical fitness and academic performance [7–9]. Poor motor proficiency at a young age is associated with behaviour that is conducive to obesity [10–14], thus a better understanding of this association to the healthcare challenge of obesity is needed.

Overweight and obesity are reported to correlate negatively with motor proficiency at various ages [10, 12, 14–16]. Overweight is furthermore considered to be a precursor of poor motor execution and this causal relationship was not found to be reversed [17]. A recent review revealed that overweight specifically affects gross motor skills, as excess body weight contributes to loss of postural control and balance [18], while in-depth studies of brain function also yielded proof of alterations in white matter organisation [10]. Researchers attribute changes in brain plasticity to excess fat accumulation and are of the opinion that it can be associated with both poorer motor control and poorer cognitive abilities [6]. An increase in body mass index (BMI) also shows significant associations with decreased motor coordination abilities in five- to 11-year-old children [14, 16, 17].

Contradicting findings showing no associations between gross motor proficiency and adiposity among three- to six-year-olds from low income rural and low- and high-income urban backgrounds in South Africa, when physical activity levels were taken into consideration, have also been reported [19].

A limited number of longitudinal studies could be found in this area in the literature. One longitudinal study did, however, reveal an association between increased body weight and decreased motor skills [12]. Weight loss between 10 and 14 years of age led to improved motor skills and allowed previous overweight children to catch up on their peers, while good motor skill abilities at baseline decreased the chances of becoming overweight at a later age [12].

Although many cross-sectional studies report an association between motor proficiency and obesity in children, information about the longitudinal relationship between these factors are still scarce and often equivocal. Longitudinal studies investigating associations between BMI status and perceptual-motor skills in the developing years of children are therefore considered to be necessary. In addition, obesity studies often concentrate on overweight children as a whole, while taking changing BMI status into account might bring a new understanding of the link between obesity and perceptual-motor skills of young children. Future research in this regard to investigate to what extent weight loss and decreased adiposity would improve overweight and obese children’s gross motor skill competence has been recommended [20]. South African obesity statistics show that children growing up in this country follow the same trend of increasing obesity as currently reported in other countries [21]. Understanding the association between persistent overweight from a young age and PMP will aid in finding early intervention solutions to combat obesity-related triggers and future obesity-related consequences. The main aim of this study was therefore to determine the longitudinal influences of persistent overweight on PMP of children residing in the North-West Province of South Africa, over the primary school years, while also studying the effects of changing weight status.

## Methods

### Study design and sampling

Primary school children in the North-West Province of South Africa served as the target population of the longitudinal study *North-West Child Health, Integrated with Learning and Development* (*NW-CHILD*). The study was conducted over the primary school period of seven school years (2010–2016). Baseline testing was conducted in 2010, followed by two follow-up time measurements 3 years apart in 2013 and 2016. Stratified random sampling was used to identify boys and girls in 2010 during their grade 1 year for the baseline measurements. Follow-up measurements took place in grade 4 in 2013 (for some participants their grade 3 year), and in grade 7 in 2016 (for some participants their grade 6 year), enabling analyses over a six-year follow-up period over seven school years. Random sampling was based on a list of schools received from the Department of Basic Education in the North-West Province, where after stratification was done according to school size, school districts, regions and school types (Quintile 1 to 5). From eight education districts, representing 12 to 22 regions with approximately 20 schools (minimum 12, maximum 47) per region, four school districts and 20 schools were randomly selected taking population density and school status into account (Quintile 1 – schools from poor economic areas, to Quintile 5 – schools from wealthier economic areas). South Africa uses a poverty classification system to categorise schools in different quintile groups regarding factors related to socioeconomic status. The classification system of the National Census information, which includes income, the ratio of dependents, as well as literacy levels, is used [22]. Schools with a Quintile 4 and 5 classification represent high socioeconomic areas, while Quintile 1 to 3 schools represent low socioeconomic areas*.

### Study population

The study population included 2010, 2013 and 2016 follow-up measurements. Recruitment of participants in 2010 included 864 subjects, of which 816 consented to be measured at baseline. The mean age of this group was 6.86 years (± 0.39) in 2010, 9.90 years (± 0.38) in 2013, and 12.9 years (± 0.38) in 2016. Complete follow-up data were available for 381 participants (46.69% of the original group). It has been reported in a previous study on the same population that no evidence of bias due to loss of subjects during the follow-up period could be found [5].

The 381 participants represented both genders (181 boys and 200 girls), quintile schools (Quintile 1 to Quintile 5), and two racial groups (white *n* = 104; black *n* = 277). Two hundred and 18 (57.22%) participants were from low socioeconomic areas (92 boys, 126 girls, 218 black). The high socioeconomic status group consisted of 163 (42.78%) participants (89 boys, 74 girls, 104 white; 59 black). Data were collected by senior researchers in Kinderkinetics and Kinderkinetics Honours students.

### Instruments and measurements

#### Anthropometry

Body weight was measured in kilogram (kg) to the nearest 0.1 kg with an Omron (BF 511) electronic scale, and height in centimeters (cm) with a Harpenden-stadiometer. Body mass index (BMI) was expressed as kg/m^2^. For the purpose of this study, the participants were classified in one of the following BMI groups: never-overweight (N-O); normal-to-overweight (N-OW); persistent-overweight (P-OW); and overweight-to-normal (OW-N). International gender- and age-specific cut-off values based on BMI percentiles for overweight and obesity were used for classification [23]. Participants were identified with overweight when their age- and gender-specific BMI fell between the 85th and 95th percentile, and for obesity when the BMI was above the 95th percentile*. For the purpose of this study, the term overweight included participants identified with overweight and obesity. The “never-overweight” group reflected BMI values below the 85th percentile during each of the three measurements, from their grade 1-year until grade 7. The “normal-to-overweight” group included participants with BMI values lower than the 85th percentile in grade 1, but with increased BMI values of above the 85th when they were in grade 7. The “persistent-overweight” group included participants that presented with BMI values above the 85th percentile at each of the three measurements from grade 1 to grade 7. The “overweight-to-normal” group included participants who presented with BMI values higher than the 85th percentile in grade 1, but who had BMI values lower than the 85th percentile in grade 7, showing a reversing trend towards normal weight.

#### Bruininks Oseretsky test of motor proficiency, second edition (BOT-2)

The *Bruininks Oseretsky Test of Motor Proficiency,* second edition (*BOT-2*) assesses proficiency in motor skills between four and 21 years of age. The test battery consists of four main components, namely fine motor control, dexterity coordination, body coordination, strength and agility, with eight sub-components, including (i) fine motor precision, (ii) fine motor integration, (iii) manual dexterity, (iv) bilateral coordination, (v) balance, (vi) running speed and agility, (vii) upper limb coordination and (viii) strength. Four assessment options are available for the *BOT-2*, namely a complete form, short form, selected components and selected sub-components. For the purpose of this study, the Short Form (*BOT-2 SF*) and three complete sub-components (strength, running speed and agility, and balance) were used. The short form took approximately 20 to 30 min to complete. Point allocation was done per sub-component, after which raw scores were converted to point scores. Point scores were totalled and converted to standard scores, which were then used to calculate gender- and age-equivalent descriptive categories and percentile values. The *BOT-2* is a reliable test battery with values that vary between *r* = 0.7 and *r* = 0.8 for different age groups, with sufficient validity to provide effective measurements of gross and fine motor skills [24], while also showing proof of a useful instrument to assess changes over time [25, 26].

### Ethical considerations

Ethical approval for the study was obtained from the Health Sciences Ethics Committee of the North-West University (NW-00070-09-A1) and the Department of Basic Education of the North-West Province, South Africa. School principals had to provide permission for data collection during school hours. The parents and/or legal guardians of all the invited participants had to provide informed parental consent, while each participant had to provide child assent before participation in the study was allowed. All the shools that were part of the research design were contacted and then visited 4 months before re-testing would occur to obtain re-consent from the headmaster. Participants that were previously part of the study were then recruited from the class lists of the school, after which parents and participants had to provide follow-up consent for participation. The research was conducted according to the declaration of Helsinki and “Ethics in Health Research: Principles, Processes and Structures” [27]. An inclusion criterion was used once the required permission had been given, if the participants were present on the day of the testing and if the data for the three time-point measurements (2010, 2013, 2016) were available. An exclusion criterion was applied when the aforementioned requirements were not met. If a participant’s first language was neither English nor Afrikaans, trained interpreters who had been pre-trained were used to explain each test accurately to the participants.

### Data analysis and statistical methods

*Statistica for Windows, Version 13.3* was used to analyse the data [28]. For descriptive purposes, the data were analysed to derive means (M), standard deviations (sd) and minimum and maximum values [28].

Repeated measures ANOVA was performed on all PMP variables for the different overweight groups over time. This analysis explored group differences at set time points, within-group differences over time and between-group differences over time. A *p*-value of ≤0.05 indicated statistical significance. Effect sizes were used to report the practical differences between PMP variables and the different BMI groups at each of the three time points, where significant differences were indicated by Cohen’s d-values of small (d = 0.2), medium (d = 0.5) and large (d = 0.8) cut-offs [29].

## Results

Table 1 provides the group characteristics while also displaying descriptive characteristics of the four different BMI groups as divided by BMI cut-offs. The group included 200 (52.49%) girls and 181 (47.51%) boys, of which 72.7% (*n* = 277) were black and 27.3% (*n* = 104) white. The socioeconomic characteristics of the group indicated that 218 (57.22%) represented low socioeconomic- and 163 (42.78%) high socioeconomic schools.

**Table 1:** Distribution of participants (*N* = 381) by BMI group

Variables	N	%	Never overweight		Normal → overweight		Persistent overweight		Overweight → normal	
			N	%	n	%	n	%	n	%
**Total group**	**381**	**100**	286	75.07	34	**8.92**	38	**9.97**	23	6.04
**Boys**	**181**	**47.51**	135	35.44	18	4.72	17	4.46	11	2.89
**Girls**	**200**	**52.49**	151	39.63	16	4.20	21	5.51	12	3.15
**White**	**104**	**27.30**	63	16.54	14	3.67	19	4.99	8	2.10
**Black**	**277**	**72.70**	223	58.52	20	5.25	19	4.99	15	3.94
**Low SES**	**218**	**57.22**	188	49.34	15	3.94	9	2.37	6	1.57
**High SES**	**163**	**42.78**	98	25.72	19	4.99	29	7.61	17	4.46

*N* number of participants; *SES* Socioeconomic status; *%* percentage

*The method of determining overweight and SES is stipulated in the Methods section

From the total of 381 participants, 9.97% (*n* = 38) were categorised as persistently overweight (P-OW) from grade 1 to grade 7. By the end of the primary school years, 18.89% (*n* = 72) of the total group were overweight, as a further 8.92% (*n* = 34) shifted to higher BMI values during the seven-year period. The additional 8.92% of participants were placed for comparison purposes in a separate category described as the normal-to-overweight group (N-OW). At the same time, 6.04% (*n* = 23) of the group who had been overweight in grade 1 reflected a decrease in their BMI and were placed in the overweight-to-normal BMI group (OW-N). In both these groups that shifted to other BMI groups, the gender distribution was relatively equal, while percentages of inclusion were slightly higher for black participants in both the groups.

Table 2 summarises the descriptive results of a repeated-measures ANOVA on the running speed and agility, strength and balance subtest scale scores, as well as the Short Form (*BOT-2* total) standard scores and percentiles for the four BMI groups over the three time-point measurements. Significant group differences, as well as BMI group by time interaction effects, are also displayed in Table 2. Significant BMI group effects, as well as main effects over time within each BMI group, were found. Statistically significant (*p* ≤ 0.05) differences between the BMI groups in all the separate PMP items that were compared (running speed and agility, strength, balance and the *BOT-2* total) are confirmed. No significant differences between the groups’ *BOT-2* totals at the different time points were found, although differences over time within the groups showed significant variation. However, no statistically significant interaction effects between BMI group and time were obtained in the sub-items, although group differences in the *BOT-2* total and strength and agility percentile, showed marginal significance (*p* = 0.053 and *p* = 0.083).

**Table 2:** Repeated measures ANOVA results of PMP differences between participants in different BMI groups over the three time-point measurements

		Perceptual-motor proficiency components													
		R and A (sc)		Strength (sc)		Balance (sc)		S and A (ss)		S and A (%)		*BOT-2* total (ss)		*BOT-2* total (%)	
		*Mean*	*95% CI*	*Mean*	*95% CI*	*Mean*	*95% CI*	*Mean*	*95% CI*	*Mean*	*95% CI*	*Mean*	*95% CI*	*Mean*	*95% CI*
**Never overweight**	**Gr. 1**	18.11	17.70–18.52	16.31	15.91–16.71	12.84	12.34–13.35	55.03	54.10–55.96	66.05	63.54–68.56	41.56	40.80–42.33	24.09	21.87–26.31
	**Gr. 4**	18.20	17.86–18.54	17.45	17.08–17.82	13.82	13.17–14.47	56.49	55.69–57.29	71.78	69.36–74.20	49.98	49.08–50.88	50.09	47.22–52.97
	**Gr. 7**	17.80	17.11–18.48	14.16	13.86–14.44	15.76	15.30–16.22	52.07	51.48–52.66	57.67	55.69–59.66	45.65	44.97–46.32	34.89	32.69–37.08
**Normal → overweight**	**Gr. 1**	18.32	17.13–19.52	15.85	14.70–17.01	12.50	10.91–14.09	54.82	52.13–57.52	67.62	60.35–74.88	41.88	39.66–44.11	24.24	17.80–30.67
	**Gr. 4**	17.15	16.16–18.13	16.56	15.48–17.64	12.00	9.95–14.05	54.29	51.98–56.61	64.94	57.94–71.94	49.06	46.45–51.67	46.09	37.75–54.43
	**Gr. 7**	18.85	16.87–20.83	13.53	12.69–14.37	13.68	12.24–15.13	49.94	48.25–51.64	48.59	42.86–54.32	44.21	42.24–46.17	30.21	23.84–36.57
**Persistent overweight**	**Gr. 1**	16.73	15.61–17.87	15.03	13.93–16.12	11.77	10.31–13.23	51.45	48.90–54.00	54.82	47.94–61.69	42.05	39.95–44.16	24.76	18.67–30.85
	**Gr. 4**	16.11	15.18–17.03	16.05	15.03–17.07	11.46	9.58–13.34	51.97	49.78–54.17	54.69	48.07–61.32	51.24	48.77–53.71	51.45	43.56–59.34
	**Gr. 7**	14.79	12.92–16.66	12.71	11.91–13.51	13.23	11.90–14.56	47.08	45.48–48.68	41.79	36.37–47.21	43.53	41.67–45.39	28.68	22.67–34.70
**Overweight → Normal**	**Gr. 1**	18.05	16.56–19.53	15.45	14.02–16.89	12.05	10.38–13.72	53.82	50.47–57.17	61.09	52.06–70.12	41.74	39.04–44.44	24.61	16.78–32.44
	**Gr. 4**	17.36	16.14–18.58	15.73	14.39–17.07	12.90	10.75–15.05	53.59	50.71–56.47	61.91	53.20–70.61	49.61	46.43–52.78	49.09	38.95–59.22
	**Gr. 7**	17.50	15.04–19.96	13.68	12.63–14.73	16.35	14.84–17.86	51.27	49.17–53.38	55.32	48.19–62.45	46.35	43.96–48.74	36.70	28.96–44.43
**MSE**		14.9		6.5		12.34		28.0		237.0		24.5		251.9	
**BMI group** (*p*-value)		< 0.001*		0.001*		0.003*		< 0.001*		< 0.001*		0.914		0.778	
**Time** (*p*-value)		0.311		< 0.001*		< 0.001*		< 0.001*		< 0.001*		< 0.001*		< 0.001*	
**BMI**^**a**^ * Time (*p*-value)		0.215		0.808		0.292		0.491		0.053		0.083		0.356	

*R and A* running speed and agility; *S and A* strength and agility; *sc* scale score; *ss* standard score; *%* percentile; *BOT-2 total* BOT-2 Short Form total; *Mean* mean value; *95% CI* 95% Confidence interval (lower – upper); *BMI* body mass index; *BMI*^*a*^ body mass index groups; *MSE* mean square error; *p ≤ 0.05

Different patterns of change in the different BMI groups over the three time points was also noted, as shown in Table 2. The P-OW group presented with the lowest scores in all the PMP test items over the three time points. A widening of differences in PMP between this group and the other groups also became especially noticeable from grade 4 to grade 7. The P-OW group did not only have the lowest running speed and agility standard scores of all the groups at all three time points, but also was the only group that showed a clear lowering trend in running speed and agility scores over the three time points. A comparison of the group mean values obtained for strength showed a similar trend. Although a decrease in strength was seen in all of the groups from grade 4 to grade 7, a larger drop was again observed in the P-OW group (16.05 to 12.71). On the contrary, balance improved within each BMI group over time, although the performance of the P-OW group was still the poorest at all time points.

Table 3 reflects the practical significance of differences that were found between PMP and the BMI groups at each of the three time points. Effect sizes indicated significant differences in running speed and agility performance at all three time-points among P-OW participants, compared to those categorised in the other three groups.

**Table 3:** Practical significant PMP differences (Cohen’s d-values) between BMI groups at each time point

		Normal-to-overweight			Persistent Overweight			Overweight-to-normal		
		Gr. 1	Gr. 4	Gr. 7	Gr. 1	Gr. 4	Gr. 7	Gr. 1	Gr. 4	Gr. 7
		*d-value*	*d-value*	*d-value*	*d-value*	*d-value*	*d-value*	*d-value*	*d-value*	*d-value*
**Never overweight**	*R and A (sc)*	0.06	0.36*	0.18	0.39*	0.72**	0.51**	0.02	0.29*	0.05
	*Strength (sc)*	0.13	0.28*	0.25*	0.37*	0.44*	0.58**	0.25*	0.54**	0.19
	*Balance (sc)*	0.09	0.37*	0.60**	0.28*	0.48*	0.74**	0.21*	0.19	0.17
	*S and A (ss)*	0.03	0.32*	0.42*	0.45*	0.66**	0.99***	0.15	0.42*	0.16
	*S and A (%)*	0.07	0.33*	0.53**	0.52**	0.82***	0.93***	0.23*	0.48*	0.14
	*BOT-2 total (ss)*	0.05	0.12	0.25*	0.07	0.16	0.36*	0.03	0.05	0.12
	*BOT-2 total (%)*	0.01	0.16	0.25*	0.04	0.05	0.33*	0.03	0.04	0.10
**Normal-to-overweight**	*R and A (sc)*	–	–	–	0.45*	0.36*	0.69**	0.08	0.07	0.23*
	*Strength (sc)*	–	–	–	0.24*	0.16	0.33*	0.12	0.26*	0.06
	*Balance (sc)*	–	–	–	0.19	0.11	0.13	0.12	0.18	0.78**
	*S and A (ss)*	–	–	–	0.42*	0.34*	0.57**	0.13	0.10	0.26*
	*S and A (%)*	**–**	**–**	**–**	0.59**	0.49*	0.40*	0.30*	0.15	0.40*
	*BOT-2 total (ss)*	–	–	–	0.03	0.28*	0.12	0.02	0.07	0.37*
	*BOT-2 total (%)*	–	–	–	0.03	0.22*	0.08	0.02	0.12	0.34*
**Persistent Overweight**	*R and A (sc)*	–	–	–	–	–	–	0.37*	0.43*	0.46*
	*Strength (sc)*	–	–	–	–	–	–	0.12	0.10	0.39*
	*Balance (sc)*	–	–	–	–	–	–	0.07	0.29*	0.91***
	*S and A (ss)*	–	–	–	–	–	–	0.30*	0.24*	0.83***
	*S and A (%)*	–	**–**	**–**	**–**	**–**	**–**	0.29*	0.35*	0.80***
	*BOT-2 total (ss)*	–	–	–	–	–	–	0.05	0.21*	0.48*
	*BOT-2 total (%)*	–	–	–	–	–	–	0.01	0.10	0.42*

*R and A* running speed and agility; *S and A* strength and agility; *BOT-2 total* BOT-2 Short form total; *sc* scale score; *ss* standard score; *%* percentile; *Practical significance of differences* d > 0.2*; 0.5**; 0.8***

Practically significant running speed and agility differences of moderate to large magnitudes were especially noticeable between the P-OW and the N-O group (d = 0.39, d = 0.51 and d = 0.72), as displayed in Table 3. The strength of the P-OW group also differed practically significantly from the N-O group (d = 0.37, d = 0.44 and d = 0.58), while the strength of the OW-N group was also moderately significantly poorer than the N-O group in their grade 4 year (d = 0.54). Balance differences between the P-OW and the N-O group were also of moderate (d = 0.48) and large (d = 0.74) practical significance at grades 4 and 7. In both these grades, the balance of the N-O group differed significantly (d = 0.37 and d = 0.60) on a practical level from the N-OW group, with the latter performing worse.

Overall, PMP (*BOT-2* total) did not differ between the BMI groups in grade 1 and grade 4, although small to medium differences emerged in the grade 7 year. These differences were once again found mostly between the P-OW group and the N-O group, while the OW-N group in grade 7 reflected practically significant better PMP than the N-OW group (d = 0.37).

## Discussion

The aim of this study was to determine if a longitudinal association existed between persistent overweight and PMP of primary school children in the North-West Province of South Africa. An overweight prevalence of 18.89% was found at the end of the primary school years. Of these, 9.97% were persistently overweight over the period of 7 years, while an additional 8.92% of the participants also became overweight by the time they were in grade 7. These findings agree with WHO statistics, where 18% of girls and 19% of boys worldwide between the ages of 5 and 19 years of age were overweight in 2016 [1]. Compared to National South African obesity statistics reported in 2012, our study shows increasing trends compared to the 11.5 and 4.7% in boys, and 16.5 and 7.1% in girls aged 2–14 years that were reported [21]. Although reversing trends were found in 6.04% of the group, almost one in 10 of the participants (9.97%) showed a persistent nature in their overweight status from grade 1 to grade 7, which is most likely not to change in the future.

Persistent overweight and obesity resulted in poor motor performance, as the P-OW group exhibited significantly poorer PMP compared to all other three BMI groups, especially in comparison to the N-O group. With increasing age, their perceptual-motor coordination even appeared to deteriorate. Clear differences were found in running speed, agility and strength between the groups, and effect sizes of differences increased over time, which portrays the practical significant longitudinal effect of persistent overweight on PMP. The N-O group showed significantly better strength and agility compared to groups that had a historic or current presence of excess body weight. Lastly, PMP differences found in the changing groups (N-OW and OW-N) also showed significance, mostly in grade 7, where either the presence or absence of excess body weight seemed to influence the results. A comparable longitudinal study on 10- to 14-year-old children [12] agrees with these findings where normal weight children outperformed their overweight peers when executing motor skills. In addition, the study found that the impact of change in weight status throughout the longitudinal period on motor skills favoured weight loss in overweight children. A large sample (*N* = 3738) of six- to 10-year-olds was categorised in different BMI groups (thin, normal, overweight, obese) to compare their motor coordination skills [14]. Aligned with our results, the normal-BMI group portrayed the best motor coordination, while the overweight and obese BMI groups showed the poorest motor coordination of all the groups [14].

The influence of overweight on specific PMP skills signifies BMI group differences in running speed and agility, strength and balance. Once again, the P-OW group generally performed poorer compared to the other groups in all these comparisons. Poorer strength, running speed and agility among obese children have also been reported by other researchers [11, 30, 31]. It has been suggested that the correlation between BMI and motor skills partly depends on whether the task requires projection of body mass through space or supporting movement where the body works against gravity [14]. These PMP skills measured in our study all require projection or movement of body mass, where these motor skill characteristics might have contributed to our findings. Similar results, specifically on sideways jumping, push-ups and a 6-min run, were reported, all requiring movement of the body through space or against gravity [12]. A study on 10-year-old girls divided into malnourished, normally nourished, pre-obese and obese groups also found that obese girls struggled significantly more with motor tests requiring quick movements of the entire body over long distances or power-based movements, including running speed, repetitive strength and explosive power of the lower extremities [32].

It is noteworthy that in the current study, balance improved within each BMI group over time, although balancing skills of the P-OW group was still the lowest at all time points. Other studies also linked poor balancing skills with obesity, although the associations were generally least pronounced and of small magnitudes [20, 30, 31]. These findings were also derived from different aged participants in studies of a cross-sectional nature. Motor skill abilities, such as balancing, walking backwards and catching, were generally of the same level in obese and normal weight children in a large population-based sample of four- to six-year-old USA children [11]. The findings of another study also revealed no significant balancing skills differences at ages five- to seven years, although differences were significant at older ages [20]. They concluded that BMI-related differences in gross motor coordination were more pronounced as children belonged to an older age group (10 to 12 years versus five to seven-year-olds), but also recommend that this finding should be investigated further in longitudinal studies [20]. Our longitudinal analysis confirmed significantly poorer balancing skills in the persistent overweight group. Therefore, when obesity persists into older ages, the influence of a heavier weight on motor skills appears to go beyond only impacting activities including weight bearing, power driven, projection through space or movement against gravity. These more pronounced differences might be the result of the continuous interrelationship between childhood overweight, physical inactivity and motor incompetence. Guo et al. have made the important statement that the age of a population does play a role and should be considered when findings regarding weight and motor skills are reported and compared [33].

The longitudinal effects of losing or gaining weight on proficiency in motor skills were also studied, which improved our understanding of the changes in the group with persistent overweight. When adiposity decreased, an improving effect on PMP was found, compared to a deteriorating effect when weight was gained over the study period. While no differences were found in the overall motor proficiency of all the groups (*BOT-2* total) in grade 1 at 6 years, the OW-N group reflected practically significant better PMP than the N-OW (d = 0.37) in grade 7 at 12 years. The group that changed from a normal to overweight status (N-OW) over the 7-year period, again performed practically significantly poorer in balancing skills than the normal-weight group in grades 4 and 7 (d = 0.37 and d = 0.60). These results should again be considered against the widening of differences between the P-OW group and all the other groups in grade 7. These results, therefore, confirm a beneficial effect of lowering weight on motor proficiency, but on the other side, also confirmed the negative effect of unhealthy weight gain on perceptual-motor skill competence. In agreement, another longitudinal study also reported that a change in weight status (weight loss in overweight children) over a 4-year period was favourable to children’s motor skills [12].

Our study is innovative in the sense that we separated children with persistent overweight from those with normal weight or those who showed changes in their weight status, while investigating longitudinal influences of increased weight on motor proficiency differences. We were also able to determine to what extent weight loss and decreased adiposity would improve overweight and obese children’s perceptual-motor skill competence. This study is to the best of our knowledge a first to compare influences of weight on PMP in this way over a longitudinal period of 6 years, which enabled us to illustrate an increasingly detrimental effect of persistent childhood overweight on PMP across developmental time.

Limitations of this study include a relatively large loss-to-follow-up of participants (53.31%), which may have limited the internal validity of the study findings. The final participant group size was, however, still considered large enough to provide sound statistical analysis. We also acknowledge that the *BOT-2* assessment tool that was used to determine PMP, was not developed in South Africa, which could have influenced the results. This study only made use of BMI cut-offs and it is recommended that future longitudinal studies also include waist circumference, waist-to-hip ratio and skinfolds when determining overweight and obesity, while also taking physical activity into account.

## Conclusions

Persistent overweight negatively impacts weight-bearing motor proficiency components, as well as balancing skills, especially in older age groups. These effects were more pronounced in children with persistent overweight compared to those who became overweight during the study period. Improving weight status, however, was found to counter these effects, which highlights the importance of timeous intervention to combat obesity at a young age. The overall pattern of results therefore suggests that excess adiposity might inhibit obese children from developing and exercising their motor skills, which again can contribute to declines in motor competence relative to healthy weight children [11]. These findings are clinically important because poor motor skill development is associated with children’s physical inactivity and poor fitness levels [34, 35]. As persistent overweight practically impacts PMP on a larger scale at older ages, it consequently sets the tone for unhealthy, sedentary adolescent years to follow.

Considering the key role of motor competence in physical activity engagement, which in turn is health protective, our results emphasise the need for early identification of motor difficulties in overweight and obese children. These children are still at an optimal age to develop their motor competence, which can prevent them from a further decrease in motor coordination over time. Intervention strategies that promote healthy body weight from an early age can also positively contribute to the PMP of primary school children and provide a good health foundation during the pre-adolescent years, to be sustained throughout adulthood. Physical Education teachers and movement specialists should apply these timeous interventions already during the early childhood years and motivate children throughout their primary school years to be healthy and active.

## Data Availability

All data generated and analyzed during this study is available upon request.

## Abbreviations

BMI: Body Mass Index
PMP: perceptual-motor proficiency
BOT-2: Bruininks Oseretsky Test of Motor Proficiency-2
N-OW: normal-to-overweight
OW-N: overweight-to-normal
P-OW: persistent-overweight
SES: Socioeconomic status
